# Supplementary material for: The relationship between professional quality of life and work environment among nurses in neonate care units
Source: PLoS One. 2025 Apr 25;20(4):e0322023. doi: 10.1371/journal.pone.0322023 (PMC12027106; doi:10.1371/journal.pone.0322023)
Supplement: S1 File — (DOCX) [file pone.0322023.s001.docx]

**Supporting Information**

**S1 Table: Participant Demographics**
This table provides an overview of the demographic characteristics of the 233 NICU nurses included in the study:

- **Age**:
  - ≤30 years: 139 (59.7%)
  - 31–40 years: 75 (32.2%)
  - 40 years: 19 (8.2%)
- **Gender**:
  - Male: 61 (26.2%)
  - Female: 172 (73.8%)
- **Education Level**:
  - Diploma: 64 (27.5%)
  - Bachelor’s: 151 (64.8%)
  - Master’s or higher: 18 (7.7%)
- **Work Experience**:
  - ≤5 years: 170 (73.0%)
  - 6–10 years: 36 (15.5%)
  - 10 years: 27 (11.6%)
- **Work Shift**:
  - Day shift: 87 (37.3%)
  - Rotating shift: 146 (62.7%)

**S2 Table: Professional Quality of Life Scores**
This table summarizes the levels of compassion satisfaction, burnout, and secondary traumatic stress among participants:

- **Compassion Satisfaction**:
  - Low: 0 (0.0%)
  - Average: 221 (94.8%)
  - High: 12 (5.2%)
- **Burnout**:
  - Low: 21 (9.0%)
  - Average: 212 (91.0%)
  - High: 0 (0.0%)
- **Secondary Traumatic Stress**:
  - Low: 35 (15.0%)
  - Average: 196 (84.1%)
  - High: 2 (0.9%)

**S3 Table: Work Environment Scores**
This table presents scores for various aspects of the NICU practice environment:

- **Overall Practice Environment**: Mean = 2.7 ± 0.3
- **Subscales**:
  - Nurse Participation in Hospital Affairs: 2.7 ± 0.3
  - Nursing Foundations for Quality of Care: 2.7 ± 0.3
  - Nurse Manager Ability, Leadership, and Support: 2.7 ± 0.4
  - Staffing and Resource Adequacy: 2.6 ± 0.4
  - Collegial Nurse-Physician Relations: 2.8 ± 0.5

**S4 Table: Correlation and Predictors**

- **Correlation Between Work Environment and ProQoL**:
  - Work Environment & Compassion Satisfaction: r = 0.747, p < 0.001
  - Work Environment & Burnout: r = -0.604, p < 0.001
  - Work Environment & Secondary Traumatic Stress: r = -0.151, p = 0.021
- **Significant Predictors**:
  - Higher education level correlated with a more favorable work environment (p < 0.05).
  - Nurses with >10 years of experience reported lower burnout levels (p < 0.05).

**S5 Methods: Statistical Analysis Details**

- **Software Used**: IBM SPSS Statistics Version 23
- **Analyses Conducted**: Descriptive statistics, Pearson correlation, Spearman’s correlation, t-tests, and one-way ANOVA
- **Significance Threshold**: p < 0.05
